# Marrying Perona Malik diffusion with Mamba for efficient pediatric echocardiographic left ventricular segmentation

**DOI:** 10.1038/s41598-025-16797-6

**Published:** 2025-09-01

**Authors:** Zi Ye, Tianxiang Chen, Fangyijie Wang, Hanwei Zhang, Lijun Zhang

**Affiliations:** 1 Institute of Intelligent Software, Guangzhou, 511400 Guangdong China; 2 University of Science and Technology of China, Hefei, 230026 Anhui China; 3 School of Medicine, University College Dublin, Dublin, D04 V1W8 Ireland; 4 Saarland University, 66123 Saarbrücken, Germany; 5 SKLCS, Institute of Software, University of Chinese Academy of Sciences, Beijing, 100190 China

**Keywords:** Left ventricular segmentation, Mamba, Mixture of experts, Pediatric echocardiography, Perona–Malik diffusion, Computer science, Information technology

## Abstract

Segmenting echocardiographic images is a crucial step in assessing heart function, as clinical indicators can be obtained by precisely delineating the left ventricle. The success of subsequent heart analyses depends entirely on the precision of this segmentation. However, echocardiography is characterized by ambiguity and heavy background noise interference, making accurate segmentation more challenging. Present methods lack efficiency and are prone to mistakenly segmenting some background noise areas, such as the left ventricular area, due to noise disturbance. To address these issues, we introduce P-Mamba, which integrates the Mixture of Experts (MoE) concept for efficient pediatric echocardiographic left ventricular segmentation. Specifically, we utilize the recently proposed ViM layers from the vision mamba to enhance our model’s computational and memory efficiency while modeling global dependencies. In the DWT-based (Discrete Wavelet Transform) Perona-Malik Diffusion (PMD) Block, we introduce a block that suppresses noise while preserving the left ventricle’s local shape cues. Consequently, our proposed P-Mamba innovatively combines the PMD’s noise suppression and local feature extraction capabilities with Mamba’s efficient design for global dependency modeling. We conducted segmentation experiments on two pediatric ultrasound datasets and a general ultrasound dataset, namely Echonet-dynamic, and achieved state-of-the-art (SOTA) results. Specifically, on the Pediatric PSAX (8959 images) and Pediatric A4C datasets (6425 images), we achieved Dice scores of 0.922 and 0.906, respectively; on the EchoNet-Dynamic dataset (19882 images), we achieved a Dice score of 0.931. Leveraging the strengths of the P-Mamba block, our model demonstrates superior accuracy and efficiency compared to established models, including vision transformers with quadratic and linear computational complexity.

## Introduction

Congenital heart diseases (CHD) pose significant health risks, necessitating precise diagnostic tools like the Echocardiogram for early detection and treatment in children^1^. Among these indices, the Left Ventricular Ejection Fraction (LVEF) is the most commonly used and vital metric for assessing systolic function^2^. The LVEF is predominantly calculated using the biplane Simpson’s standard protocol method in clinical settings^3^. This technique involves the manual delineation of the left ventricular endocardium by physicians in specific frames of the apical two-chamber (A2C) and apical four-chamber (A4C) echocardiographic views, a process essential for the identification of the end-systolic volume (LVESV) and end-diastolic volume (LVEDV).

Machine learning and artificial intelligence have significantly improved the reliability and precision of segmenting the left ventricle (LV) in adult echocardiography, as shown by multiple research projects. However, machine learning is more difficult in youngsters due to diverse anatomical anomalies, heart rate, stature, and cooperative capacity. Various factors influence the spatial and temporal resolutions, eventually influencing echocardiographic imaging quality^4^. For example, the size and shape of the heart in children fluctuate considerably due to the growth and developmental stages. This variability, along with disorders such as CHD, makes pediatric heart anatomy more complicated than that of adults, limiting the application of adult-based machine learning models. Furthermore, pediatric echocardiograms frequently exhibit lower resolution and increased speckle noise, especially when obtained using portable ultrasound instruments in non-specialized settings, affecting both the spatial and temporal resolutions of the images^5^. As a result, there is concern about how well machine learning models built on adult datasets can be applied in pediatric echocardiography owing to the more significant variabilities.

State Space Models (SSMs) have recently extracted broad interest from researchers. SSMs effectively model dynamic systems using linear equations, leading to reduced computational complexity in processing sequential data. They enhance data processing speed and model scalability by integrating input-dependent mechanisms with hardware-aware architectures. In contrast to conventional Recurrent Neural Networks (RNNs) and Long Short-Term Memory (LSTM) models, SSMs optimize computational resource usage while effectively capturing long-range dependencies, making them ideal for implementation on edge devices^6^. As the first proposed basic model built by SSMs, Mamba^7^ has achieved superior performance compared to transformers in long-range dependency modeling, even with linear complexity. Vision Mamba^8^ was later proposed to apply Mamba to the vision domain and achieves superb efficiency-accuracy balance compared with DeiT. U-Mamba^9^ was the first Mamba-based medical image segmentation method and boasted its efficient design. Based on the above works, we apply the Mamba structure to our model to guarantee model efficiency.

**Fig. 1:**
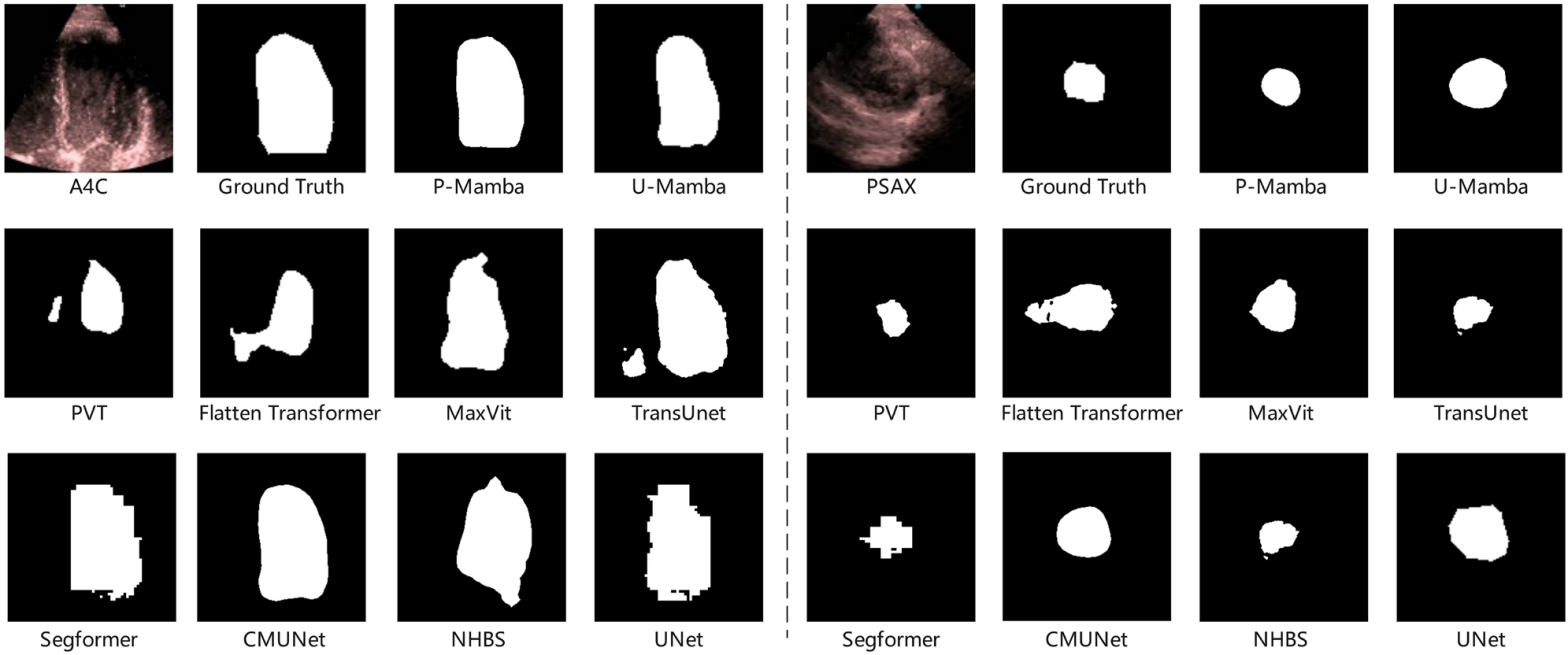
Visualization of the noise interference challenge among P-Mamba and other models on the pediatric A4C and PSAX dataset.

On the other hand, echocardiography often encounters challenges, including significant speckle noise, limiting its imaging technique. As shown in Fig. 1, current approaches easily mistake some background noise areas for the target area. Recent studies^10^ have explored denoising techniques to improve ultrasound image quality, focusing on reducing noise and enhancing visualization. To suppress background noise while maintaining target structural features, we draw inspiration from Perona–Malik Diffusion (PMD)^11^, initially in de-noising tasks to achieve this goal. Therefore, we tailor P-Mamba for more efficient and accurate pediatric echocardiographic LV segmentation. This model can eliminate noise while preserving the local target boundary details for the best performance. At the same time, our model demonstrates superior efficiency.

**Fig. 2:**
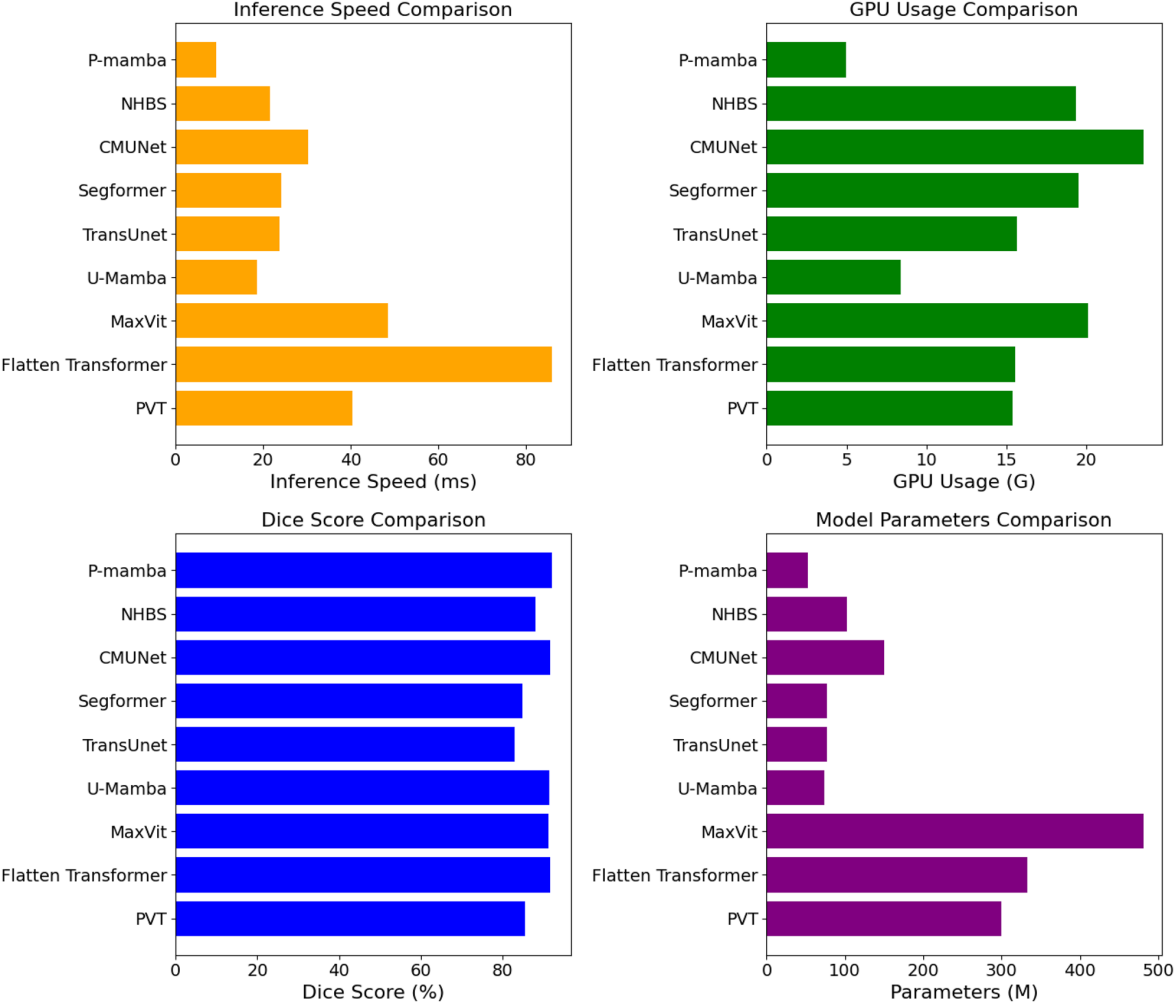
The efficiency comparisons between P-Mamba and other models on the pediatric PSAX dataset.

Our main contributions are as follows:

- We propose P-Mamba, which innovatively combines the PMD’s noise suppression and local feature extraction ability with the Vision Mamba’s efficient design for global dependency modeling and integrates the Mixture of Experts (MoE)^12^ concept to set new performance benchmarks on noisy pediatric echocardiogram datasets.
- Benefiting from PMD, our model excels by suppressing noise while preserving and enhancing target edges in ultrasound images, as shown in Fig. 1.
- Extensive experiments demonstrate that our P-Mamba achieves superior segmentation accuracy and efficiency to other methods, including specialized ultrasound segmentation models^13,14^, vision transformers with quadratic^15^ and linear^16,17^ computational complexity. In addition, Fig. 2 visually compares the efficiency among different models.

## Related work

To date, deep learning (DL) development has promoted automatic medical image segmentation, and several well-known deep learning frameworks have provided innovative ideas for echocardiography segmentation with outstanding performance.

### General deep learning segmentation methods

Deep learning has revolutionized image segmentation in recent years, a crucial task in computer vision. Among the pioneering methods, U-Net^18^ stands out for its effective encoder-decoder architecture, which captures context through its contracting path and refines localization through its expansive path. U-Net’s simplicity and efficiency have made it a foundational medical image segmentation model.

Pyramid Vision Transformer (PVT)^15^ and TransUNet^19^ both draw significant inspiration from transformer architecture. PVT captures long-range dependencies through self-attention mechanisms and hierarchical feature representations, making it highly effective for dense prediction tasks. Similarly, TransUNet integrates transformer modules into the traditional U-Net structure, leveraging the transformer’s attention mechanisms to enhance feature encoding and significantly improve segmentation accuracy, particularly in complex scenarios. Both models demonstrate the transformative impact of incorporating transformer principles into segmentation tasks.

UniFormer^20^, SpectFormer^21^, and Segformer^22^ represent some of the most contemporary segmentation models. Segformer achieves state-of-the-art performance in multiple segmentation benchmarks with lower computing costs by integrating hierarchical transformers with effective feature fusion approaches. This simplifies the transformer architecture while maintaining its key benefits. UniFormer merges convolutional networks with transformers to balance local feature extraction and global context understanding, efficiently capturing multi-scale features to enhance segmentation performance. SpectFormer delves deeper into the transformer domain by incorporating spectral analysis, emphasizing frequency domain information to complement traditional spatial representations. These models exemplify the cutting-edge advancements in segmentation technology.

### Medical image segmentation methods

Recently, innovative deep-learning methods have become increasingly vital in medical analysis and represent the forefront of leveraging complex neural networks for precise medical image segmentation. For instance, CMU-Net^13^ and NHBS-Net^14^ are specialized ultrasound segmentation models. CMU-Net uses hybrid convolution and multi-scale attention gates to improve feature extraction and context information. NHBS-Net has advanced attention and fusion modules, making clinical ultrasound applications more accurate and error-free.

U-Mamba^9^ was the first general-purpose biomedical image segmentation method which integrates the global context understanding from Mamba. It designed a hybrid CNN-SMM block and employed the encoder-decoder framework within a U-shaped structure. Recently, there have been limited Mamba-based architectures that have shown promise in the echocardiographic segmentation task. MambaEviScrib^23^ introduces Mamba blocks alongside evidence-guided consistency to improve CNN robustness in a weakly supervised learning approach. However, such methods do not explicitly address feature-space denoising, which is a general problem in ultrasound images.

In the context of biomedical image segmentation, nnU-Net^24^ has emerged as a self-configuring solution that automatically adapts to a variety of datasets without requiring manual modifications. This model streamlines the segmentation pipeline, from preprocessing to postprocessing, by using a set of heuristic rules and dataset-specific configurations. 3D nnU-Net^25^, an extension of nnU-Net, utilizes 3D convolutions to handle volumetric data, making it particularly effective in processing echocardiographic images, where maintaining temporal consistency during cardiac cycles is essential. Additionally, a post-processing approach is introduced to guarantee temporal consistency in segmentation results^26^, which employs a 2D+time cardiac shape autoencoder that corrects inconsistencies across frames by learning a physiologically interpretable embedding of the cardiac shape. This technique ensures that the segmented sequences maintain both anatomical accuracy and temporal coherence, resulting in more reliable segmentation throughout the entire cardiac cycle.

However, when applying the present segmentation methods to segment the left ventricle in echocardiograms, whether pediatric or adult, the challenge exists since the irregular shape of the left ventricles still cannot be well segmented since these methods pay insufficient attention to high-frequency boundary details, which can be seen in Fig. 1. Also, present methods lack efficiency, hindering their wider application, as shown in Fig. 2. Another concern of the studies above is the limitations of their reliance on proprietary datasets. The EchoNet-Peds dataset, developed by Stanford University, indicates the first publicly available pediatric echocardiography dataset^27^, featuring 4467 echocardiograms from 1,958 patients, including a 43$\%$ female demographic and ages ranging from newborns to 18 years. This comprehensive dataset yielded 7643 video clips and 17,600 labeled images, primarily from A4C and Parasternal Short-Axis (PSAX) view clips. As a result, the video clips were strategically allocated, with 6114 (80$\%$) for training, 765 (10$\%$) for testing, as well as 764 (10$\%$) for validation.

## Methodology

**Fig. 3:**
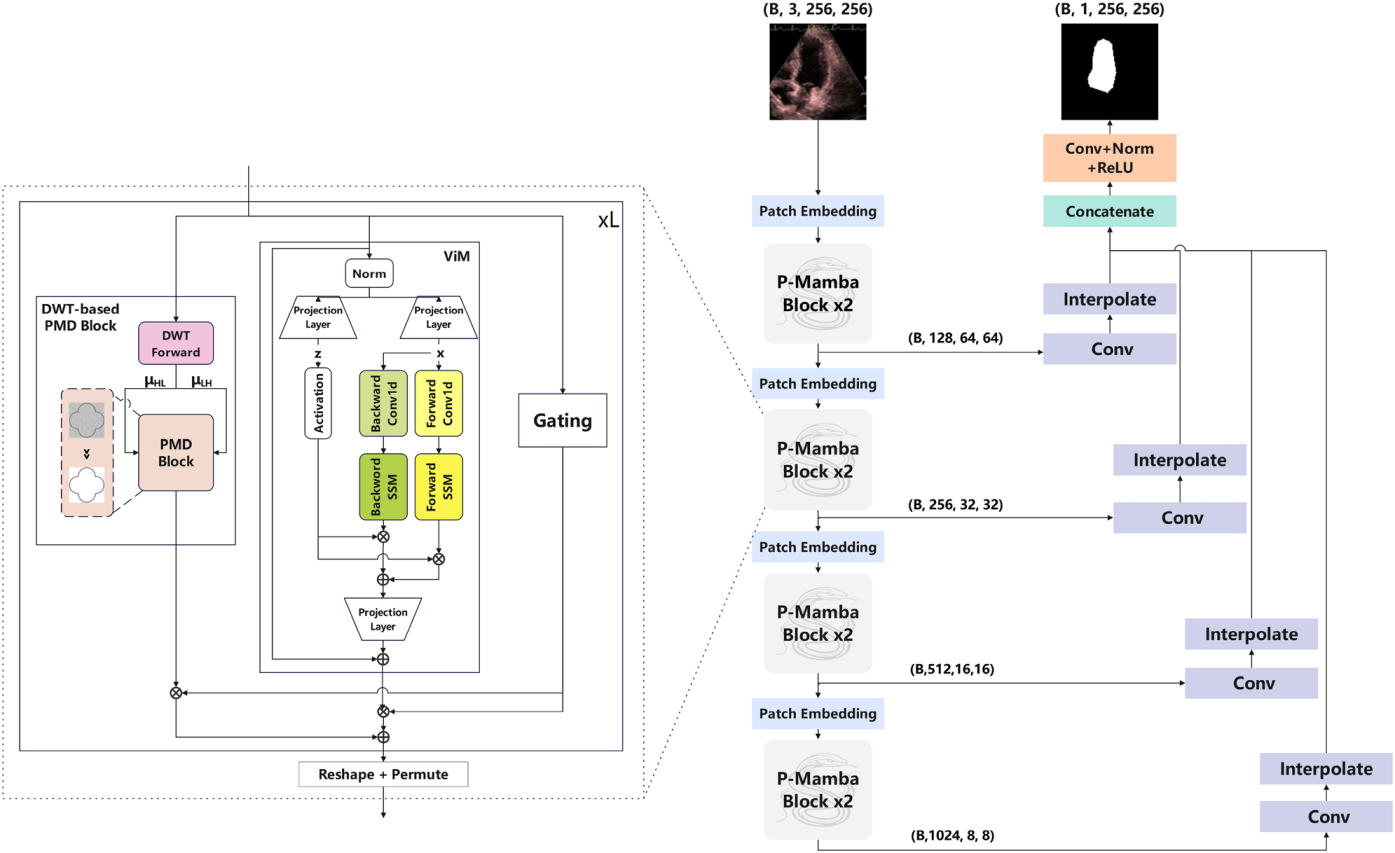
The structure of our P-Mamba. The P-Mamba block utilized the Mixture of Experts framework to leverage the DWT-based PMD block and ViM block.

We illustrate the overall architecture of P-Mamba in Fig. 3. The P-Mamba network, designed for automatically segmenting the left ventricle in echocardiograms, leverages the Mixture of Experts framework. The DWT-based PMD block suppresses background noise interference while preserving target edges to capture more local features. The ViM block^8^, sometimes also mentioned as the Bidirectional Mamba block, is designed to guarantee the high efficiency of our model while encapsulating global dependencies. A gating mechanism inspired by the MoE structure dynamically selects appropriate pathways for feature processing, enhancing efficiency and segmentation accuracy.

The P-Mamba encoder, incorporating multiple P-Mamba blocks, extracts hierarchical features at descending resolutions of 1/4, 1/8, 1/16, and 1/32 relative to the original input size while progressively increasing the channels at each level. There are mainly three reasons that Perona-Malik Diffusion is applied to the latent feature space rather than the input images. Firstly, denoising at the input level may irreversibly remove fine details essential for downstream tasks. Secondly, embedding PMD within the network can gradually reduce noise in stages based on learnt representations. Lastly, feature maps in deeper layers provide better semantic information, which preserves the boundary information better when the PMD filters out the noise. In the following sections, we will introduce the detailed structure of P-Mamba.

### DWT-based PMD block

Discrete Wavelet Transform (DWT) is a mathematical transform used to compress the feature map or signal and represent the frequency components information in the time (or space) domains. It breaks the input into several wavelets localized in both time (or space) and frequency, thus enabling multi-resolution analysis. Therefore, DWT is beneficial when local and global features need to be captured, like in applications of image compression, denoising, feature extraction in deep learning, etc^28^.

It has been noticed that the PMD is widely used in image denoising due to its ability to suppress noise disturbance while preserving boundary details. In particular, PMD models have shown that they can effectively enhance edge-aware denoising performance in feature-specific regions^29^. Motivated by this, we integrate a PMD-based module into the latent space to leverage its boundary-preserving characteristics for more robust heart ultrasound image segmentation.

Considering that echocardiograms contain heavy background noise that may interfere with segmentation accuracy, we propose the DWT-based PMD Block to act on feature maps so that the background noise can be filtered. At the same time, some target boundary cues can be preserved.

Given an input feature map *u*, its PMD equation can be expressed as:

1 $$\begin{aligned} \frac{\partial u}{\partial t}=div\left( g\left( |\nabla u| \right) \nabla u \right) \end{aligned}$$

where $g(|\nabla u|) = \frac{1}{1 + \left( \frac{|\nabla u|}{k} \right) ^2}$ is the diffusion coefficient; *t* is the diffusion step and can be regarded as the layer of the feature map; *k* is a positive constant to control the degree of diffusion and is set to 1 by default in our experiments. Notably, (1) is an anisotropic diffusion equation. In the flat or smooth regions where the gradient magnitude is small ($|\nabla u|\rightarrow 0$), the diffusion coefficient *g* is large, meaning that the diffusion is strong and (1) acts as Gaussian smoothing to remove the noise interference. Somewhere near the target’s boundary, the gradient magnitude is large ($|\nabla u|\rightarrow 1$), so the coefficient *g* is near zero, meaning the diffusion is weak, and the boundary details can be preserved. Equation (1) can also be rewritten to the following form:

2 $$\begin{aligned} \begin{aligned} \frac{\partial u}{\partial t}=&\frac{\partial }{\partial x}\left\{ g\left( \sqrt{\left( \frac{\partial u_k}{\partial x}\right) ^2+\left( \frac{\partial u_k}{\partial y}\right) ^2}\right) \frac{\partial u_k}{\partial x}\right\} \\&+\frac{\partial }{\partial y}\left\{ g\left( \sqrt{\left( \frac{\partial u_k}{\partial x}\right) ^2+\left( \frac{\partial u_k}{\partial y}\right) ^2}\right) \frac{\partial u_k}{\partial y}\right\} \end{aligned} \end{aligned}$$

where $\frac{\partial u}{\partial x}$ and $\frac{\partial u}{\partial y}$ represent the gradients of the feature map in horizontal and vertical directions. On the other hand, the Discrete Wavelet Transform (DWT) of an input feature map can be expressed as:

3 $$\begin{aligned} u_{i}=DWT(u), i \in \left\{ u_{LL},u_{LH},u_{HL},u_{HH}\right\} \end{aligned}$$

where $u_{LL}$ is the low-frequency part of the feature map, while $u_{LH}$, $u_{HL}$ and $u_{HH}$ are the high-frequency parts in horizontal, vertical and diagonal directions of the feature map which mainly contain the boundary details. By approximating the derivative terms $\frac{\partial u}{\partial x}$ with $u_{LH}$ and $\frac{\partial u}{\partial y}$ with $u_{HL}$ and setting the diffusion step size $\delta t$ to one, we can transform (2) to the discrete format:

4 $$\begin{aligned} \begin{aligned} u_k=&u_{k-1}+\left[ g\left( \sqrt{u_{L H}^2+u_{H L}^2}\right) \cdot u_{L H}\right] _{LH}\\&+\left[ g\left( \sqrt{u_{L H}^2+u_{H L}^2}\right) \cdot u_{H L}\right] _{HL} \end{aligned} \end{aligned}$$

After enhancing the feature map by PMD, we feed the diffusion output into a basic ResNet block. Piling multiple DWT-based PMD blocks in all layers of the encoder branch, our P-Mamba can suppress background noise disturbance while preserving the target boundary features.

### Vision Mamba block

Mamba^7^ is a novel deep sequence model architecture addressing the computational inefficiency of traditional Transformers on long sequences. It is based on selective state space models (SSMs), which improve upon previous SSMs by allowing the model parameters to be input functions.

Consider a structured SSM mapping one-dimensional sequence $x(t) \in \mathbb {R}^L$ to $y(t) \in \mathbb {R}^L$ through a hidden state $h(t) \in \mathbb {R}^N$. With the evolution parameter $\varvec{A}\in \mathbb {R}^{N \times N}$ and the projection parameters $\varvec{B}\in \mathbb {R}^{N \times 1}$, $\varvec{C}\in \mathbb {R}^{1 \times N}$, such a model is formulated using linear ordinary differential equations

5 $$\begin{aligned} \begin{aligned} h'(t) =&~ \varvec{A}h(t) + \varvec{B}x(t), \\ y(t) =&~ \varvec{C}h(t). \end{aligned} \end{aligned}$$

Moreover, to enhance GPU utilization and efficiently materialize the state *h* within the memory hierarchy, hardware-aware state expansion is enabled by selective scan. By incorporating kernel fusion and recomputation with parallel scan, the fused selective scan layer can effectively decrease the quantity of memory I/O operations, leading to a significant acceleration compared to conventional implementations.

We mainly adopt the ViM block to improve our model’s computing and memory efficiency. The ViM block adds a bidirectional sequence modeling (SSM) approach to better model global context to allow the model to recognise relationships across patch levels of an image. After splitting the input image into smaller patches, each was linearly projected into a vector space. These patches are treated as sequence data in the ViM block, where the bidirectional SSM efficiently models long-range dependencies and compresses the visual information. The position embedding before the P-Mamba Block adds a crucial spatial context to each patch, ensuring the model captures positional information for accurate visual recognition. This innovative approach enables the ViM block to be both computationally efficient and memory-efficient while maintaining high performance. Most importantly, it helps capture global dependencies complementary to the local shape cues extracted by our DWT-based PMD Block.

Before processing into the P-Mamba block, a 2-D input $\in \mathbb {R}^{H \times W\times C}$ is transformed into flattened patches $P_N$ with dimensions $M \times (N^2 \cdot C)$. Here (*H*, *W*) is the size of the original input, *C* stands for the number of channels, and *N* and *M* denote the size and total count of segmented patches, respectively. Then, the $P_N$ is linearly projected to vectors of dimension D, and the position embedding $E_{\text {pos}} \in \mathbb {R}^{M \times D}$ is added. This process can be described as follows:

6 $$\begin{aligned} X_0 = [x^1W; x^2W; \dots ; x^MW] + E_{\text {pos}} \end{aligned}$$

where $x^M$ is the $M^{th}$ patch of $P_N$, $W \in \mathbb {R}^{(N^2 \cdot C) \times D}$ is the learnable projection matrix. The token sequence from the patch embedding layer, $X_{pe}$, is processed by the layer ViM to obtain the output $X_{vim}$, which is expressed by (7).

7 $$\begin{aligned} X_{vim} = Vim(X_{pe}) + X_{pe} \end{aligned}$$

Subsequently, the outputs from the DWT-based PMD module and the ViM module are iteratively combined using the gating mechanism across several layers within the P-Mamba block. The final output is then reshaped and permuted to serve as the input for the next stage, where the process is repeated to obtain the next stage’s output.

### Mixture of experts

We use a lightweight mixture-of-experts design to integrate the PMD and ViM modules, and the mathematical formula is shown below:

8 $$\begin{aligned}{\bf H}_t = \text {DropPath}\left( w \cdot \text {PMD}(\textbf{H}_{t-1}) + (1 - w) \cdot \text {ViM}(\textbf{H}_{t-1})\right) \end{aligned}$$

where *w* is between 0 and 1, and it is a gating weight based on global average pooled attributes. This approach enables the network to dynamically assess the significance of structure preservation (PMD) and semantic representation (ViM).

### Decoder

The decoder consists of multiple interpolation layers interspersed with Conv2d operations, progressively upsampling the encoded features to generate high-resolution segmentation maps. Specifically, the decoder processes the output feature map from the different encoder stages through several Conv2d layers, followed by interpolation operations that align the feature maps to a common resolution. These feature maps are then concatenated and passed through a series of Conv2d, batch normalization, and ReLU layers to produce the final segmentation map.

## Experiment

### Datasets

#### Pediatric dataset

The dataset, EchoNet-Peds, comprises echocardiographic evaluations from patients at Lucile Packard Children’s Hospital Stanford from 2014 to 2021, authorized by the Stanford University Institutional Review Board. The dataset contains 4467 echocardiograms collected from 1958 patients, 43$\%$ female, aged between 0 and 18 years (mean ± SD: 10 ± 5.4 years). The patients were classified into two groups based on their echocardiographic results: those with structurally normal hearts and average ejection fraction (EF) and those with structurally normal hearts but systolic dysfunction (including dilated cardiomyopathy, chemotherapy-induced systolic dysfunction) without congenital heart disease^27^.

After additional processing, the dataset was employed to obtain apical four-chamber (A4C) and parasternal short-axis (PSAX) video clips, totaling 7643 video clips and 17600 annotated pictures. The video clips were partitioned into training (80$\%$, n=6114), testing (10$\%$, n=765), and validation (10$\%$, n=764) sets for machine learning purposes. In addition, 86$\%$ of the trials had an ejection fraction (EF) equal to or greater than 55$\%$.

#### EchoNet-dynamic dataset

To ensure the model’s effectiveness on general echocardiogram datasets, not just pediatric ones, we also conducted experiments on the EchoNet-Dynamic dataset^30^. EchoNet-Dynamic, obtained from Stanford University Hospital, is the largest publicly available dataset of apical four-chamber (A4C) cardiac echocardiograms. It includes 10030 echocardiogram clips and 9989 video samples that remained after data cleansing. Each video was examined solely for the end-diastolic and end-systolic frames. As a result, 96 images were excluded due to poor-quality ground truth, leaving 14846 out of 19882 images for the training set. The remaining images were divided into validation (2563 images) and testing (2473 images) sets. End-diastolic and end-systolic frames from the same individuals were grouped for the experiments.

### Implementation details

The computational setup consists of a single Tesla V100-32GB GPU, a 12-core CPU, and 61GB of RAM. The system operates on an Ubuntu 18 environment with CUDA 11.0 and Pytorch 1.13 software.

The network was trained for 50 epochs, beginning with an initial learning rate 1e-4, a weight decay of 0.01. Specifically, the input resolution is fixed at 256 x 256. Batch sizes of 24 were selected for training to obtain a compromise between computational efficiency and model accuracy. The model’s performance was assessed every five epochs, and early halting with a patience parameter of 10 was implemented to prevent overfitting. The network architecture was organized with layers configured in depth [2, 2, 2, 2]. During inference, all methods are benchmarked using the same test resolution and batch size to maintain consistency in runtime and memory usage measurements.

It is worth mentioning that we trained nnU-Net from scratch on our echocardiographic dataset, utilizing its auto-configuration pipeline for patch size, architecture, and data normalization.

## Results and discussion

Table 1 offers a quantitative comparison of our P-Mamba with various state-of-the-art methods on the pediatric LV 2D segmentation task and includes experiments on the general ultrasound dataset EchoNet-Dynamic. The comparison includes CNN-based methods like U-Net^18^ with FCN and PSPNet backbones, as well as ViT-based approaches such as PVT^15^, Flatten Transformer^16^, and MaxVit^17^. Moreover, we also compared Uniformer^20^ and SpectFormer^21^, both newly introduced segmentation models from the last year, showcasing advanced capabilities in image segmentation. U-Mamba^9^ represents a straightforward plan Mamba model characterized by a U-shaped structure designed specifically for effective segmentation tasks. TransUnet^19^ and Segformer^22^ are recognized as classic segmentation models, and they are widely used in the field due to their robust performance. Additionally, CMUNet^13^ and NHBS^14^ are specialized models tailored for ultrasound image segmentation, addressing the unique challenges of medical imaging.

**Table 1:** Comparison with state-of-the-art methods on precision, recall, dice, and Hausdorff distance (HD).

Methods	Pediatric PSAX				Pediatric A4C				EchoNet-dynamic			
	Precision	Recall	Dice	HD[1]	Precision	Recall	Dice	HD	Precision	Recall	Dice	HD
UNet (FCN)^18^	0.849	0.876	0.862	4.30	0.828	0.834	0.831	4.35	0.884	0.877	0.881	4.41
UNet (PSPNet)^18^	0.865	0.870	0.868	4.25	0.820	0.861	0.840	4.30	0.881	0.890	0.885	4.35
PVT^15^	0.873	0.837	0.855	4.57	0.867	0.837	0.852	4.61	0.917	0.904	0.910	4.57
UniFormer^20^	0.910	0.913	0.907	3.52	0.897	0.892	0.892	3.55	0.896	0.916	0.906	3.50
SpectFormer^21^	0.913	0.906	0.916	3.43	0.908	0.904	0.901	3.45	0.920	0.923	0.921	3.40
Flatten Transformer^16^	0.922	0.912	0.917	3.35	**0.913**	0.883	0.898	3.40	0.922	0.928	0.925	3.35
MaxVit^17^	0.902	**0.925**	0.914	3.45	0.891	0.907	0.899	3.50	0.925	0.928	0.926	3.44
U-Mamba^9^	0.928	0.902	0.914	3.45	0.902	0.886	0.894	3.50	**0.933**	0.916	0.924	3.45
TransUnet^19^	0.856	0.806	0.830	5.02	0.827	0.839	0.833	5.05	0.926	0.899	0.908	5.02
Segformer^22^	0.865	0.835	0.849	4.80	0.848	0.809	0.828	4.85	0.892	0.889	0.890	4.80
CMUNet^13^	0.920	0.913	0.916	3.41	0.907	0.896	0.902	3.45	0.930	0.927	0.928	3.41
NHBS^14^	0.873	0.890	0.882	4.53	0.886	0.872	0.879	4.55	0.922	0.916	0.919	4.52
nnUNetV2^24^	0.915	0.902	0.910	3.60	0.896	0.891	0.881	3.65	0.919	0.923	0.920	3.60
Ours	**0.932**	0.913	**0.922**	**3.31**	0.905	**0.907**	**0.906**	**3.35**	0.919	**0.945**	**0.931**	**3.30**

Significant values are in bold.

As a result, Table 1 demonstrates the superiority of our P-Mamba model. It achieves the highest average Dice Similarity Coefficient (DSC) on the PSAX and A4C pediatric datasets, with values of 0.922 and 0.906, respectively. Furthermore, our model also excels on the EchoNet-Dynamic dataset, achieving a DSC of 0.931. These results highlight the effectiveness and robustness of our approach across different datasets, outperforming the listed state-of-the-art methods.

This comparative study proved that our approach is effective in heart ultrasound images. US images tend to present a high noise level, complicating the segmentation task. The Perona-Malik Diffusion (PMD) module presented in this paper overcomes this limitation by enabling more accurate segmentation of images, as it is capable of noise reduction while still highlighting the important boundaries of the image. Additionally, we adopted ViM, a novel module that is not a conventional transformer module, it enhances the computational and memory efficiency of our model by retaining global information. More interestingly, we combine the PMD and ViM modules in a Mixture of Experts (MoE) configuration with a Neural Network. The topology also has a gating mechanism that enables it, to some extent, to follow the principles of a Mixture of Experts. This technique allows for the identification of the most suitable feature processing pathways to be selected during the processing of the features. In addition to improving the efficiency of feature processing, this approach effectively elevates the segmentation accuracy, even in the most difficult segmentation context, like echocardiogram images.

### Ablation studies

We first ablate the DWT-based PMD Block in Table 2 across various configurations: ’Ours w/o PMD’ means removing the DWT-based PMD part; ’Ours w/ Sobel’ means replacing the DWT-based PMD part with a Sobel operator, which is only for edge preservation. Our DWT-based PMD block achieves the best performance on those three datasets. The DWT-based PMD part encompasses the Sobel operator due to its noise suppression and edge preservation capability, while the Sobel operator cannot finely preserve edges during noise removal.

**Table 2:** Ablation study on the DWT-based PMD block design.

Methods	PSAX dataset			A4C dataset			EchoNet-dynamic		
	Precision	Recall	Dice	Precision	Recall	Dice	Precision	Recall	Dice
Ours w/o PMD	0.890	0.910	0.900	0.880	0.881	0.880	0.922	0.893	0.907
Ours w/ Soble	0.931	0.901	0.916	**0.907**	0.899	0.903	**0.924**	0.917	0.920
Ours	**0.932**	**0.913**	**0.922**	0.905	**0.907**	**0.906**	0.919	**0.945**	**0.931**

Significant values are in bold.

In addition, Table 3 studies the effect of the Vision Mamba block. We replaced the ViM block with ViT structures of quadratic (PVT) and linear complexity (Flatten ViT, MaxViT). The results, summarized across the PSAX, A4C, and EchoNet-Dynamic datasets, indicate that Vision Mamba consistently achieves a higher Dice coefficient. Our findings indicate that Vision Mamba can be more accurate than other models with linear or quadratic complexity.

**Table 3:** Ablation study on the ViM block design.

Methods	PSAX dataset			A4C dataset			EchoNet-dynamic		
	Precision	Recall	Dice	Precision	Recall	Dice	Precision	Recall	Dice
Mamba $\rightarrow$ PVT	0.903	**0.918**	0.911	0.905	0.871	0.888	0.915	0.891	0.903
Mamba $\rightarrow$ Flatten Transformer	0.914	0.916	0.915	**0.923**	0.875	0.898	0.920	0.897	0.909
Mamba $\rightarrow$ MaxVit	0.925	0.909	0.917	0.906	0.901	0.904	**0.921**	0.900	0.911
Ours	**0.932**	0.913	**0.922**	0.905	**0.907**	**0.906**	0.919	**0.945**	**0.931**

Significant values are in bold.

To further validate the effectiveness of the mixture of expert’s gating network convergence, we compared it with the results of simply adding the outputs of the DWT-based PMD Block and the ViM module. Table 4 shows that the gating structure improves the Dice coefficient across three datasets, demonstrating that the gating structure implemented with a linear layer significantly improves precision.

**Table 4:** Ablation study on the converge design.

Methods	PSAX dataset			A4C dataset			EchoNet-dynamic		
	Precision	Recall	Dice	Precision	Recall	Dice	Precision	Recall	Dice
Adding	0.920	**0.920**	0.920	**0.909**	0.883	0.896	**0.932**	0.927	0.930
Ours (Gating)	**0.932**	0.913	**0.922**	0.905	**0.907**	**0.906**	0.919	**0.945**	**0.931**

Significant values are in bold.

### Model efficiency comparison

Table 5 presents the model efficiency comparison results of P-Mamba with various state-of-the-art methods, considering parameters, inference speed (ms), GPU memory usage (GB), and GFLOPs. Our P-Mamba significantly outperforms other methods across all metrics. Specifically, P-Mamba has the lowest parameter count (52.77M), fastest inference speed (9.18 ms), least GPU memory usage (4.95 GB), and lowest GFLOPs (24.66). The attention-free design of our Mamba block greatly enhances model efficiency, even compared to models with linear complexity. Additionally, our DWT-based PMD block does not add excessive parameters compared to the pure mamba net like U-Mamba, ensuring high efficiency.

**Table 5:** Model efficiency comparison regarding parameter number, inference speed, GPU memory, and GFLOPs.

Methods	#Params (M)	Inference time (ms)	GPU memory (GB)	GFLOPs
PVT^15^	299.81	40.39	15.39	181.88
Flatten Transformer^16^	333.43	86.12	15.55	184.08
MaxVit^17^	480.79	48.49	20.08	259.48
U-Mamba^9^	74.00	18.64	8.38	37.14
TransUnet^19^	77.08	23.82	15.67	36.69
Segformer^22^	77.20	24.07	19.48	38.90
CMUNet^13^	149.93	30.22	23.54	182.68
NHBS^14^	102.20	21.64	19.35	119.49
Ours	**52**.**77**	**9**.**18**	**4**.**95**	**24**.**66**

Significant values are in bold.

### Qualitative comparison

**Fig. 4:**
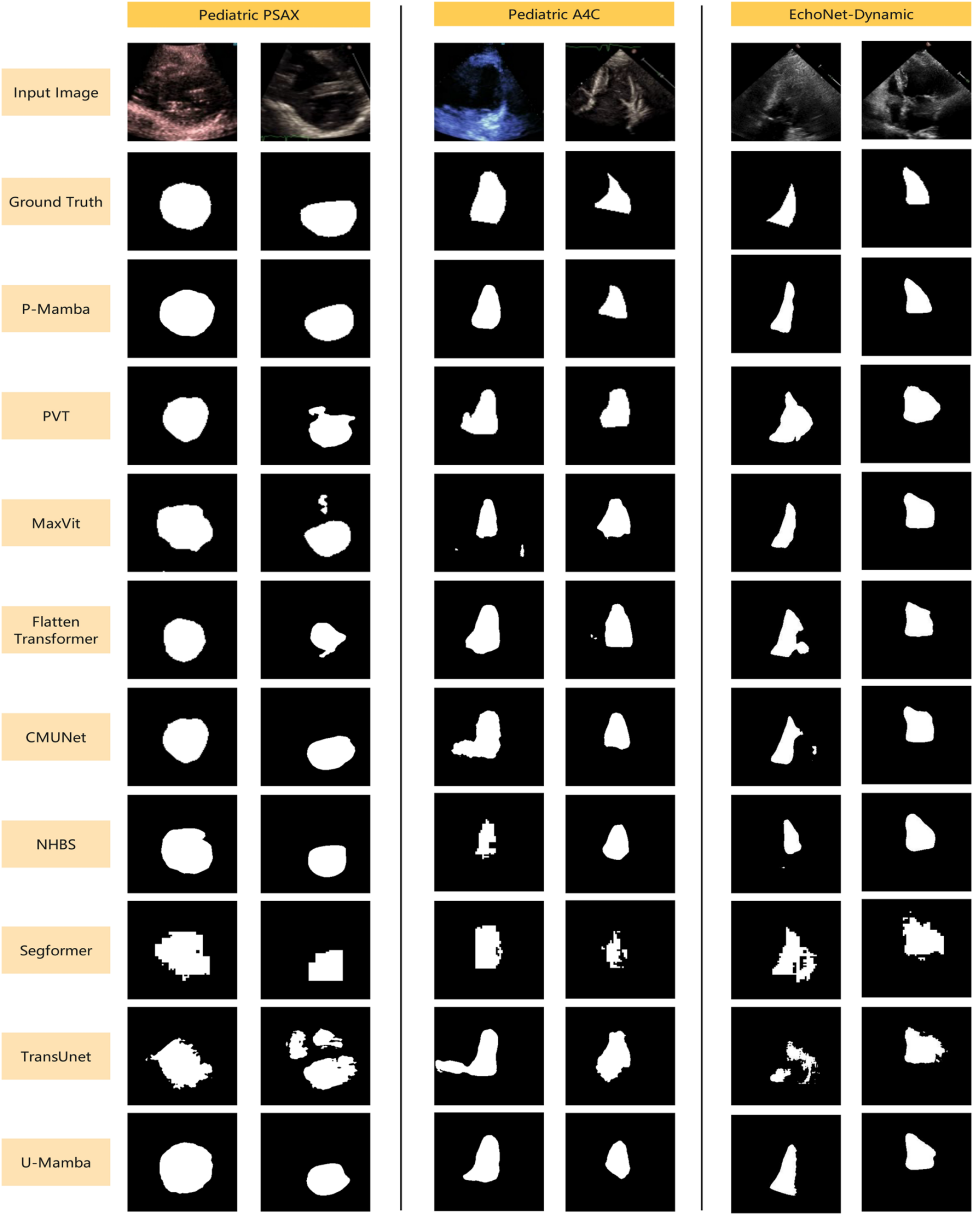
The visual comparison of different methods.

The qualitative results in Fig. 4 illustrate the performance of various models on pediatric PSAX, pediatric A4C, and EchoNet-Dynamic image segmentation tasks. Classic segmentation models like Segformer and TransUnet display poor segmentation results, indicating their unsuitability for echocardiogram data modalities. Due to noise interference, PVT, Flatten Transformer, MaxVit, and U-Mamba struggle to delineate heart structures correctly. Specialized ultrasound segmentation models such as NHBS and CMUNet also fall short in accurately segmenting the A4C views. In contrast, our P-Mamba is the least affected by noise interference and enjoys the best segmentation effect, thanks to our PMD design.

**Fig. 5:**
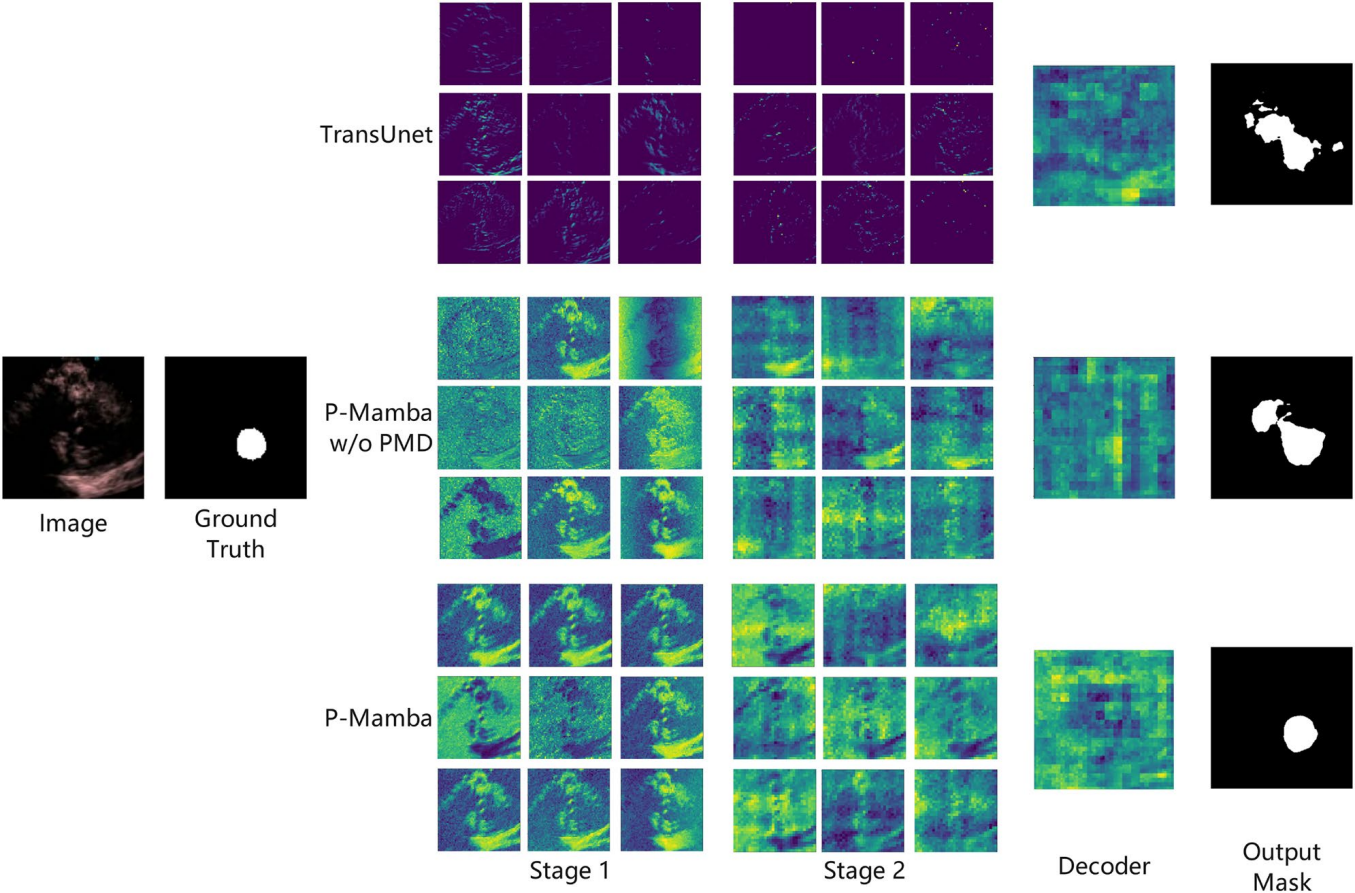
The Visualization of the features of TransUnet, P-Mamba w/o PMD, and P-Mamba. The P-Mamba has shown more concentrated feature contours.

We visualize the learned features of TransUnet, P-Mamba without the DWT-based PMD Block, and P-Mamba to better understand the impact of the proposed method. The feature map outputs of encoder stages 1 and 2 are shown in Fig. 5, with nine feature map channels randomly sampled for each method. With more distinct feature contours, local information is better preserved in P-Mamba than in other methods. Additionally, we visualize the feature map output of the final decoder stage in Fig. 5 to support our analysis. We observe that P-Mamba features are more concentrated and exhibit stronger discriminative power.

## Limitation and future work

One limitation of this study is that our experiments were focused exclusively on segmenting the left ventricle in echocardiographic images without exploring its application to other cardiac structures, such as myocardial segmentation^31^. Expanding the evaluation of additional anatomical regions, particularly the myocardium, would provide a more comprehensive understanding of the method’s performance across various heart components. Furthermore, automated Left Ventricular Ejection Fraction (LVEF) measurement based on this segmentation model can be included to enable heart functional assessment, since it is a critical metric in clinical cardiac evaluation

Additionally, the applicability of our approach to other ultrasound imaging modalities and tasks has yet to be investigated. Future research could investigate the generalization of our method to other types of ultrasound images, such as those used in abdominal or musculoskeletal imaging, to evaluate its robustness and versatility.

As large-scale models advance, an interesting direction for future research would be to investigate whether our method adheres to the scaling laws observed in deep learning^32^, particularly concerning model size, data quantity, and computational requirements. Exploring these aspects could provide valuable insights into the scalability and potential of our approach in more complex or larger datasets.

## Conclusion

We present P-Mamba, a model tailored for efficient left ventricular segmentation in pediatric echocardiography, overcoming the background noise interference challenge. Specifically, the DWT-based Perona-Malik Diffusion Block is a mathematically explainable module focusing on local feature extraction. It can gradually suppress background noise disturbance while preserving the boundary details of the left ventricle. The vision Mamba block, on the other hand, can explore global dependencies. We integrate the two parts by borrowing the Mixture of Experts (MOE) ideas, and our P-Mamba successfully achieves an exceptional balance between segmentation accuracy and efficiency.

## Data Availability

All data used in this study were obtained from publicly available databases: https://stanfordaimi.azurewebsites.net/datasets/834e1cd1-92f7-4268-9daa-d359198b310a and https://stanfordaimi.azurewebsites.net/datasets/a84b6be6-0d33-41f9-8996-86e5df53b005

## Code availability

The code for P-Mamba is available at https://github.com/yezizi1022/P-Mamba and can be cited using the DOI: https://zenodo.org/records/14899330.
